# Single-step ethanol production from lignocellulose using novel extremely thermophilic bacteria

**DOI:** 10.1186/1754-6834-6-31

**Published:** 2013-02-28

**Authors:** Vitali A Svetlitchnyi, Oliver Kensch, Doris A Falkenhan, Svenja G Korseska, Nadine Lippert, Melanie Prinz, Jamaleddine Sassi, Anke Schickor, Simon Curvers

**Affiliations:** 1 Direvo Industrial Biotechnology GmbH, Nattermannallee 1, D-50259, Köln, Germany

**Keywords:** Anaerobic, *Caldicellulosiruptor*, Consolidated bioprocessing, Ethanol, Extremely thermophilic bacteria, High temperature, Lactate, Lignocellulose, *Thermoanaerobacter*

## Abstract

**Background:**

Consolidated bioprocessing (CBP) of lignocellulosic biomass to ethanol using thermophilic bacteria provides a promising solution for efficient lignocellulose conversion without the need for additional cellulolytic enzymes. Most studies on the thermophilic CBP concentrate on co-cultivation of the thermophilic cellulolytic bacterium *Clostridium thermocellum* with non-cellulolytic thermophilic anaerobes at temperatures of 55°C-60°C.

**Results:**

We have specifically screened for cellulolytic bacteria growing at temperatures >70°C to enable direct conversion of lignocellulosic materials into ethanol. Seven new strains of extremely thermophilic anaerobic cellulolytic bacteria of the genus *Caldicellulosiruptor* and eight new strains of extremely thermophilic xylanolytic/saccharolytic bacteria of the genus *Thermoanaerobacter* isolated from environmental samples exhibited fast growth at 72°C, extensive lignocellulose degradation and high yield ethanol production on cellulose and pretreated lignocellulosic biomass. Monocultures of *Caldicellulosiruptor* strains degraded up to 89-97% of the cellulose and hemicellulose polymers in pretreated biomass and produced up to 72 mM ethanol on cellulose without addition of exogenous enzymes. In dual co-cultures of *Caldicellulosiruptor* strains with *Thermoanaerobacter* strains the ethanol concentrations rose 2- to 8.2-fold compared to cellulolytic monocultures. A co-culture of *Caldicellulosiruptor* DIB 087C and *Thermoanaerobacter* DIB 097X was particularly effective in the conversion of cellulose to ethanol, ethanol comprising 34.8 mol% of the total organic products. In contrast, a co-culture of *Caldicellulosiruptor saccharolyticus* DSM 8903 and *Thermoanaerobacter mathranii* subsp. *mathranii* DSM 11426 produced only low amounts of ethanol.

**Conclusions:**

The newly discovered *Caldicellulosiruptor* sp. strain DIB 004C was capable of producing unexpectedly large amounts of ethanol from lignocellulose in fermentors. The established co-cultures of new *Caldicellulosiruptor* strains with new *Thermoanaerobacter* strains underline the importance of using specific strain combinations for high ethanol yields. These co-cultures provide an efficient CBP pathway for ethanol production and represent an ideal starting point for development of a highly integrated commercial ethanol production process.

## Background

Ethanol is an established alternative fuel from renewable resources [1]. Today it is mainly produced from sugar or starchy biomass, limiting the environmental benefit [2] and posing a competition for the raw materials with food industry. In the last decade research efforts have mounted to replace this 1st generation ethanol by the 2nd generation ethanol made from lignocellulosic feedstocks, including pretreatment, enzymatic hydrolysis, sugar fermentation and process design. Most of the processes developed toward industrial scale involve addition of enzymes for cellulose and hemicellulose hydrolysis and use of specific yeast strains engineered to utilize C5 and C6 sugars. Both achieving effective biomass hydrolysis and complete sugar conversion are essential for an economical process. Although enzyme producers have made substantial improvements in the recent years, cost of cellulase enzymes are still in the range of $0.5 to $1.0 per gallon of 2nd generation ethanol [3,4].

A process strategy that aims to circumvent this critical cost-increasing item is the consolidated bioprocessing approach [3,5]. In CBP an organism or a mixed culture of organisms produces enzymes for hydrolysis of cellulose and hemicellulose in lignocellulosic biomass and ferments the C5 and C6 sugars into ethanol or other valuable products without addition of cellulolytic or hemicellulolytic enzymes. Several mesophilic and thermophilic cellulolytic as well as non-cellulolytic microorganisms with engineered cellulase activity are under development for the application in CBP [3,6].

Until now, the most well developed candidate for thermophilic CBP is the anaerobic thermophilic cellulolytic bacterium *Clostridium thermocellum*[7-9]. Because *C. thermocellum* is unable to ferment C5 sugars [7], co-cultures with C5 sugar fermenting thermophilic ethanologenic bacteria of the genera *Thermoanaerobacterium*[8,10,11] and *Thermoanaerobacter*[12,13] have been developed to increase ethanol yield from cellulose and hemicellulose. Co-cultures of engineered *C. thermocellum* and *Thermoanaerobacterium saccharolyticum* produced 38.1 g/l ethanol from Avicel (crystalline cellulose), which is the highest ethanol concentration reported for a thermophilic, cellulolytic co-culture to date [8]. However, the performance of these monocultures and co-cultures was not evaluated with real lignocellulosic substrates under industrial conditions and few data have been reported yet for *C. thermocellum* co-cultures that would support the process viability using pretreated and untreated lignocellulosic substrates under laboratory conditions [11,13].

Realization of CBP at extremely high temperatures (>70°C) would offer several advantages over mesophilic and thermophilic (50°C-60°C) conditions: increased stability of enzymes and organisms, decreased medium viscosity, no requirement for cooling, elimination of pathogenic bacteria, low risk of contamination and facilitated continuous product recovery [13,14].

Extremely thermophilic (temperature optimum for growth >70°C) cellulolytic bacteria of the genus *Caldicellulosiruptor* and non-cellulolytic bacteria of the genera *Thermoanaerobacterium* and *Thermoanaerobacter* were also studied as potential CBP organisms. *Caldicellulosiruptor* species effectively hydrolyze both cellulose and hemicelluloses, metabolize C5 and C6 sugars and can grow on pretreated as well as on untreated lignocellulosic materials like switchgrass and poplar [15-18]. They produce lactate, acetate, H_2_ and CO_2_, whereas ethanol is detected only in trace amounts [15-17]. Because of very low ethanol production by known *Caldicellulosiruptor* species, they have so far been investigated primarily for conversion of lignocellulose to H_2_[15]. However, because of their high growth temperature and hydrolytic capabilities *Caldicellulosiruptor* species have a high potential in production of ethanol from lignocellulose. *Thermoanaerobacterium* and *Thermoanaerobacter* species [19-21] hydrolyze hemicellulose (e.g. xylan) and ferment C5 and C6 sugars to ethanol as major fermentation product. For *T. saccharolyticum*, a genetic tool to produce ethanol at high yield was recently developed [19].

Here we present the results of screening for extremely thermophilic bacteria enabling direct conversion of lignocellulosic biomass to ethanol at >70°C. We demonstrate ethanol production by newly isolated *Caldicellulosiruptor* and *Thermoanaerobacter* strains on various pretreated lignocellulosic materials in monocultures and established dual co-cultures.

## Results

### Enrichment of extremely thermophilic bacteria for conversion of lignocellulosic substrates to ethanol

For cellulolytic enrichments, media with filter paper strips and untreated beech wood as substrate were inoculated with more than 200 environmental samples. After incubation at 72°C for 7 days under anaerobic conditions, numerous cultures displayed decomposition of filter paper. For the enrichment of cellulolytic bacteria resistant against inhibitors present in pretreated lignocellulosic biomass [22], the cultures were grown on media containing unwashed dilute acid steam-explosion-pretreated poplar wood followed by serial dilutions. From 39 cellulolytic enrichment cultures obtained from the highest dilutions, 11 cultures produced substantial amounts of ethanol upon growth on filter paper as well as on steam-explosion-pretreated poplar wood (Additional file 1: Table S1). These cultures were selected for isolation of ethanologenic bacteria.

### Composition of ethanologenic enrichment cultures

**Figure 1:**
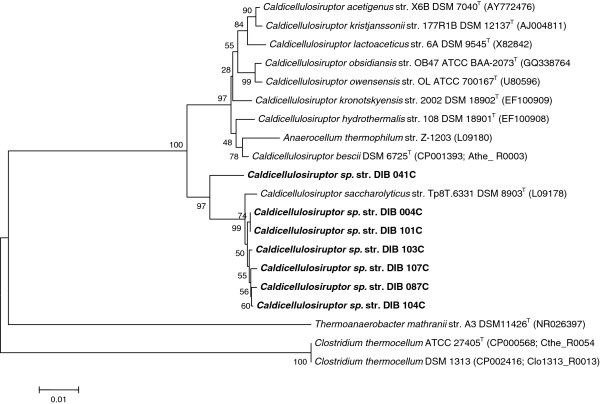
**Phylogenetic tree of *Caldicellulosiruptor *strains. **Neighbor-joining tree based on 16S rRNA gene sequence comparisons of isolated *Caldicellulosiruptor *sp. strains and selected bacteria. Bootstrap values are based on 1,000 replicates. The scale bar represents 0.01 changes per nucleotide position. GenBank accession numbers are given in parentheses. T, type strain.

Seven strains of cellulolytic bacteria and eight strains of non-cellulolytic bacteria were isolated from the ethanologenic enrichments. Based on 16S rRNA gene sequence analysis, all cellulolytic strains belonged to the genus *Caldicellulosiruptor* (Figure 1): str. DIB 004C, DSM 25177, GenBank accession number JX988415; str. DIB 041C, DSM 25771, GenBank accession number JX988416; str. DIB 087C, DSM 25772, GenBank accession number JX988417; str. DIB 101C, DSM 25178, GenBank accession number JX988418; str. DIB 103C, DSM 25773, GenBank accession number JX988419; str. DIB 104C, DSM 25774, GenBank accession number JX988420; str. DIB 107C, DSM 25775, GenBank accession number JX988421. The closest relative of all isolates was *Caldicellulosiruptor saccharolyticus* str. Tp8T.6331 (DSM 8903) [23] with 16S rRNA gene sequence identity ranging from 96.7% (strain DIB 041C) to 99.3% (strains DIB 004C, DIB 101C and DIB 103C).

**Figure 2:**
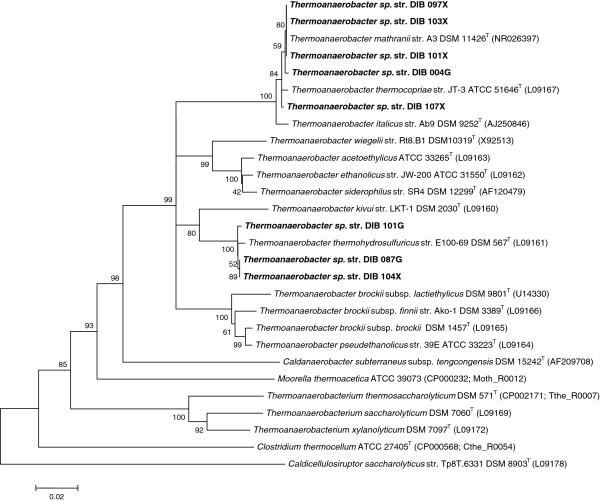
**Phylogenetic tree of *Thermoanaerobacter *strains. **Neighbor-joining tree based on 16S rRNA gene sequence comparisons of isolated *Thermoanaerobacter *sp. strains and selected bacteria. Bootstrap values are based on 1,000 replicates. The scale bar represents 0.01 changes per nucleotide position. GenBank accession numbers are given in parentheses. T, type strain.

All isolated non-cellulolytic strains were members of the genus *Thermoanaerobacter* (Figure 2): str. DIB 004G, DSM 25179, GenBank accession number JX988422; str. DIB 087G, DSM 25777, GenBank accession number JX988423; str. DIB 101G, DSM 25180, GenBank accession number JX988425; str. DIB 097X, DSM 25308, GenBank accession number JX988424; str. DIB 101X, DSM 25181, GenBank accession number JX988426; str. DIB 103X, DSM 25776, GenBank accession number JX988427; str. DIB 104X, DSM 25778, GenBank accession number JX988428; str. DIB 107X, DSM 25779, GenBank accession number JX988429. The closest relative of the strains DIB 004G, DIB 097X, DIB101X, DIB 103X and DIB 107X was the xylanolytic bacterium *Thermoanaerobacter mathranii* subsp. *mathranii* str. A3 (DSM 11426T) [20] with identity range of 99.2% to 99.4%. Strains DIB 101G, DIB 087G and DIB 104X clustered with the xylanolytic bacterium *Thermoanaerobacter thermohydrosulfuricus* str. E100-69 (DSM 567T) [21] with identities of 99.4%-99.5%.

All isolated *Caldicellulosiruptor* sp. strains grew well at 72°C on crystalline cellulose (Avicel and filter paper), cellobiose, glucose, xylan and xylose, forming lactate, acetate and ethanol as organic fermentation products. When grown in Hungate tubes, lactate was generally the main fermentation product, followed by acetate and ethanol (Table 1 and Additional file 1: Table S2). In this respect, the isolated strains were similar to *C. saccharolyticus* DSM 8903 used as a control (Table 1).

**Table 1:** Fermentation products of cellulolytic and non-cellulolytic strains isolated from ethanologenic enrichment cultures

**Enrichment cultures used for isolation**	**Substrate**	**Isolated cellulolytic *Caldicellulosiruptor *sp. strains**					**Isolated non-cellulolytic *Thermoanaerobacter *sp. strains**				
		**Strain**	**Ethanol mM**	**Acetate mM**	**Lactate mM**	**Ethanol:Acetate:Lactate mM:mM:mM**	**Strain**	**Ethanol mM**	**Acetate mM**	**Lactate mM**	**Ethanol:Acetate:Lactate mM:mM:mM**
#4	Cellulose (26 mM gluc.equiv.)	DIB004C	1.6 ± 0.7	4.8 ± 0.5	9.6 ± 0.1	1 : 3.0 : 6.1	DIB004G	no growth on cellulose			
	Cellobiose (25 mM gluc. equiv.)		0.3 ± 0.2	8.6 ± 0.1	18.8 ± 0.1	1 : 29.7 : 64.7		21.6 ± 0.6	2.4 ± 0.1	17.1 ± 0.1	1 : 0.1 : 0.8
	Glucose (25 mM)		1.2 ± 0.1	6.4 ± 0.2	19.6 ± 0.5	1 : 5.2 : 15.9		23.2 ± 0.2	0.7 ± 0.1	17.9 ± 0.7	1 : 0.0 : 0.8
	Xylan (31 mM xylose equiv.)		1.5 ± 0.2	9.6 ± 1.0	13.2 ± 3.1	1 : 6.5 : 9.0		no growth on xylan			
	Xylose (30 mM)		3.2 ± 0.4	2.5 ± 0.9	18.6 ± 0.1	1 : 0.8 : 5.9		17.1 ± 0.4	0.0 ± 0.0	10.3 ± 0.0	1 : 0.00 : 0.6
#97	Cellulose (26 mM gluc.equiv.)	no cellulolytic strains isolated					DIB097X	no growth on cellulose			
	Cellobiose (25 mM gluc. equiv.)							29.2 ± 0.2	5.3 ± 1.0	8.9 ± 0.3	1 : 0.2 : 0.3
	Glucose (25 mM)							31.7 ± 0.7	2.8 ± 0.4	11.9 ± 0.0	1 : 0.1 : 0.4
	Xylan (31 mM xylose equiv.)							19.8 ± 0.6	9.0 ± 0.1	5.1 ± 0.0	1 : 0.5 : 0.3
	Xylose (30 mM)							25.5 ± 2.0	1.1 ± 0.3	8.2 ± 1.5	1 : 0.0 : 0.3
#101	Cellulose (26 mM gluc.equiv.)	DIB101C	1.1 ± 0.1	6.9 ± 0.1	6.6 ± 0.6	1 : 6.4 : 6.2	see Additional file 1: Table S2 for further details				
	Cellobiose (25 mM gluc. equiv.)		2.6 ± 0.5	11.1 ± 0.1	14.8 ± 0.1	1 : 4.2 : 5.6					
	Glucose (25 mM)		1.5 ± 0.0	9.7 ± 0.6	12.7 ± 0.5	1 : 6.6 : 8.6					
	Xylan (31 mM xylose equiv.)		2.2 ± 0.2	10.0 ± 0.3	9.4 ± 0.2	1 : 4.5 : 4.3					
	Xylose (30 mM)		5.1 ± 0.8	4.8 ± 0.7	10.5 ± 0.0	1 : 0.9 : 2.1					
Control strains	Cellulose (26 mM gluc.equiv.)	*Caldicellulosiruptor saccharolyticus *DSM 8903	1.4 ± 0.3	5.7 ± 0.1	6.6 ± 0.1	1 : 4.1 : 4.7	*Thermoanaerobacter mathranii *DSM11426	no growth on cellulose			
	Cellobiose (25 mM gluc. equiv.)		4.7 ± 0.3	7.1 ± 1.5	14.4 ± 2.0	1 : 1.5 : 3.0		12.4 ± 0.3	7.2 ± 0.5	18.7 ± 1.2	1 : 0.6 : 1.5
	Glucose (25 mM)		1.7 ± 0.2	7.8 ± 1.1	14.2 ± 3.7	1 : 4.7 : 8.6		17.2 ± 0.4	8.5 ± 0.5	17.0 ± 1.3	1 : 0.5 : 1.0
	Xylan (31 mM xylose equiv.)		1.8 ± 0.3	10.2 ± 0.4	7.7 ± 0.3	1 : 5.5 : 4.2		19.9 ± 0.7	4.9 ± 0.4	2.7 ± 0.0	1 : 0.2 : 0.1
	Xylose (30 mM)		2.9 ± 0.5	4.1 ± 0.8	14.7 ± 1.3	1 : 1.4 : 5.1		13.7 ± 0.0	4.9 ± 0.1	12.6 ± 0.6	1 : 0.4 : 0.9

Cultures were grown at 72°C for 6 days in Hungate tubes without shaking. Experiments were performed in duplicates.

All isolated *Thermoanaerobacter* sp. strains did not utilize cellulose, grew well at 72°C and differed in their ability to utilize xylan (Table 1 and Additional file 1: Table S2). Saccharolytic strains DIB 004G, DIB 087G and DIB 101G grew on cellobiose, glucose and xylose, but not on xylan. Xylanolytic strains DIB 097X, DIB 101X, DIB 103X, DIB 104X and DIB 107X grew on cellobiose, glucose, xylan and xylose. On all substrates tested, isolated *Thermoanaerobacter* sp. strains generated ethanol, lactate and acetate as organic fermentation products. When grown in Hungate tubes ethanol was the main fermentation product of strains DIB 004G, DIB 097X, DIB 101G, DIB 101X, DIB 103X and DIB 107X (Table 1 and Additional file 1: Table S2). Lactate was the main fermentation product of strain DIB 087G. Strain DIB 104X produced almost equal amounts of ethanol and lactate. In this respect, DIB 104X was similar to the well known ethanologenic bacterium *T. mathranii* DSM 11426 used as a control (Table 1).

### Growth on pretreated washed lignocellulosic substrates

Natural lignocellulosic biomass (e.g. wood, straw, grass) requires high-temperature pretreatment to make insoluble carbohydrate polymers accessible to hydrolytic enzymes. However, Yang et al. demonstrated growth of *Caldicellulosiruptor bescii* (formerly *Anaerocellum thermophilum*) on untreated poplar and switchgrass [17].

We have tested the ability of cellulolytic *Caldicellulosiruptor* strains DIB 004C and DIB 101C to ferment insoluble carbohydrates from dilute sulfurous acid steam-explosion-pretreated substrates (Table 2 and Additional file 1: Tables S3 and S4). To remove soluble carbohydrates, pretreated substrates (poplar, spruce, miscanthus, wheat straw, whole corn plants, corn cobs, corn stalks, sugarcane bagasse, sweet sorghum, cotton stalks) were washed extensively with hot water. The remaining insoluble material was used as the sole fermentable substrate for cultivation of the bacteria. Strains DIB 004C and DIB 101C grew well on all these materials and produced lactate, acetate and ethanol indicating utilization of insoluble carbohydrates present in the washed substrates.

**Table 2:** **Fermentation products of *Caldicellulosiruptor *DIB004C alone and in co-cultures with *Thermoanaerobacter *DIB004G and DIB097X**

**Culture**	**Substrate**	**Fermentation products**					
		**Ethanol mM**	**Acetate mM**	**Lactate mM**	**Ethanol:Acetate:Lactate mM:mM:mM**	**Total products mM**	**Ethanol yield mol%**
DIB004C	Avicel (10 g/l)	2.7 ± 0.2	21.4 ± 0.1	9.0 ± 0.1	1 : 8.0 : 3.4	33.0 ± 0.0	8.1 ± 0.5
DIB004G		no growth					
DIB097X		no growth					
DIB004C+DIB004G		8.8 ± 0.7	19.9 ± 0.1	11.0 ± 0.6	1 : 2.3 : 1.3	39.7 ± 1.1	22.1 ± 1.3
DIB004C+DIB097X		10.3 ± 0.7	18.6 ± 0.7	11.8 ± 0.6	1 : 1.8 : 1.1	40.7 ± 0.8	25.4 ± 1.1
DIB004C	washed pretreated poplar (2.9 g/l)	1.5 ± 0.1	10.9 ± 0.2	6.2 ± 0.8	1 : 7.2 : 4.1	18.6 ± 0.8	8.1 ± 1.0
DIB004G		no growth					
DIB097X		no growth					
DIB004C+DIB004G		5.7 ± 0.3	14.0 ± 0.2	6.6 ± 0.9	1 : 2.5 : 1.2	26.3 ± 1.4	21.7 ± 0.1
DIB004C+DIB097X		3.7 ± 0.2	14.1 ± 0.4	8.3 ± 1.3	1 : 3.9 : 2.3	26.1 ± 1.5	14.0 ± 1.6
DIB004C	unwashed pretreated poplar (10 g/l)	2.6 ± 0.2	12.0 ± 0.2	6.1 ± 0.2	1 : 4.6 : 2.3	20.7 ± 0.2	12.6 ± 1.0
DIB004G		4.3 ± 0.3	4.4 ± 0.6	3.0 ± 0.1	1 : 1.0 : 0.7	11.7 ± 0.4	37.0 ± 3.8
DIB097X		5.1 ± 0.1	4.3 ± 0.3	0.0 ± 0.0	1 : 0.8 : 0.0	9.4 ± 0.2	54.5 ± 2.2
DIB004C+DIB004G		5.7 ± 0.4	11.6 ± 0.1	3.1 ± 0.0	1 : 2.0 : 0.5	20.4 ± 0.4	27.9 ± 1.2
DIB004C+DIB097X		7.0 ± 0.1	11.4 ± 0.2	3.5 ± 0.4	1 : 1.6 : 0.5	21.8 ± 0.6	32.1 ± 0.6

Cultures were grown at 72°C for 6 days in flasks with shaking at 100 rpm. Experiments were performed in duplicates.

Table 2 shows the fermentation products of strain *Caldicellulosiruptor* DIB 004C upon growth on medium with 2.9 g/l of washed pretreated poplar. This material contained 58.1% glucan, 0.1% xylan, 0.2% galactan, 2.7% acid-soluble lignin, 34.8% acid-insoluble lignin, and 0.1% lignin ash, as determined by standard laboratory methods [24]. At 2.9 g/l of washed pretreated poplar, the medium contained 10.4 mM glucose, 0.02 mM xylose, and 0.03 mM galactose equivalents. Assuming a theoretical yield of 2.0 mol of lactate+acetate+ethanol per mol of glucose or galactose and 1.67 mol of lactate+acetate+ethanol per mol of xylose, formation of a maximum of 20.9 mM lactate+acetate+ethanol could be expected. After 6 days of incubation, strain DIB 004C produced 6.2 mM lactate, 10.9 mM acetate and 1.5 mM ethanol (18.6 mM lactate+acetate+ethanol) which corresponded to utilization of 89.1% of insoluble cellulose and hemicellulose in pretreated poplar (Table 2).

Similar to washed pretreated poplar, high levels of utilization of insoluble cellulose and hemicellulose fractions in pretreated washed spruce, miscanthus, wheat straw, whole corn plants, corn cobs, corn stalks, sugarcane bagasse, sweet sorghum and cotton stalks (all at 2.9 g/l) were observed with cellulolytic *Caldicellulosiruptor* strains DIB 004C and DIB 101C (Additional file 1: Table S4). The highest levels of carbohydrate utilization were reached on spruce (97%), corn cobs (97%) and corn stalks (93%).

The non-cellulolytic strains *Thermoanaerobacter* DIB 004G and DIB 097X did not grow on washed pretreated poplar (Table 2) and other washed pretreated substrates confirming complete removal of soluble carbohydrates by the applied washing procedure.

### Growth on pretreated unwashed substrates

The ability to ferment pretreated unwashed lignocellulosic substrates was investigated with *Caldicellulosiruptor* DIB 004C and *Thermoanaerobacter* DIB 004G and DIB 097X. The bacteria grew well on 10 g/l of all unwashed dilute sulfurous acid steam-explosion-pretreated substrates tested (poplar, spruce, miscanthus, wheat straw, whole corn plants, corn cobs, corn stalks, sugarcane bagasse, sweet sorghum, cotton stalks), as well as on untreated dried distillers grains with solubles (DDGS) and waste paper (only DIB 004C on the last substrate). Growth was obvious from the increase of turbidity and pressure, decrease of pH and production of ethanol, lactate and acetate (Table 2 and Additional file 1: Table S3).

Total concentrations of fermentation products formed by non-cellulolytic strains DIB 004G and DIB 097X were generally lower than the concentrations achieved with the cellulolytic strain DIB 004C (Table 2 and Additional file 1: Table S3) indicating utilization of cellulose by strain DIB 004C in pretreated materials. Similar to growth on defined substrates (Table 1), ethanol production by strain DIB 004C on pretreated substrates in flasks without pH control was relatively low (7 to 13 mol% ethanol from organic fermentation products lactate+acetate+ethanol), whereas ethanol production by ethanologenic DIB 004G and DIB 097X was high (23 to 63 mol% ethanol), see Table 2 and Additional file 1: Table S3.

### Ethanol production by co-cultures of *Caldicellulosiruptor* and *Thermoanaerobacter* strains on lignocellulosic substrates grown without pH control in flasks

Cellulolytic and non-cellulolytic ethanologenic strains were isolated from ethanologenic enrichments (Table 1 and Additional file 1: Table S2) indicating coexistence of these organisms in their natural habitats. Extracellular hydrolysis of cellulose by *Caldicellulosiruptor* strains in these enrichments obviously supports the growth of non-cellulolytic ethanologenic *Thermoanaerobacter* strains resulting in natural cellulolytic ethanologenic co-cultures.

**Figure 3:**
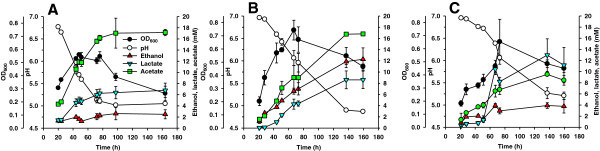
**Growth and fermentation products on Avicel. **Cellulolytic strain *Caldicellulosiruptor *sp. DIB 087C (**A**), co-culture of DIB 087C and the non-cellulolytic strain *Thermoanaerobacter* sp. DIB 097X (**B**) and co-culture of *Caldicellulosiruptor saccharolyticus *DSM 8903 and *Thermoanaerobacter mathranii *DSM 11426 (**C**) were grown on 10 g/l Avicel in flasks without pH control at 72°C and 100 rpm. Each data point represents the mean ± standard deviation calculated from samples collected from three independent experiments.

We employed several of the isolated *Caldicellulosiruptor* and *Thermoanaerobacter* strains to establish defined dual co-cultures and tested their potential for ethanol production from Avicel and several lignocellulosic substrates (Figure 3, Table 2 and Additional file 1: Table S3). Different co-cultures were analyzed under batch conditions in flasks without pH control.

Table 2 and Additional file 1: Table S3 show fermentation products upon growth of single cellulolytic strain DIB 004C, single non-cellulolytic strains DIB 004G or DIB 097X as well as co-cultures DIB 004C + DIB 004G and DIB 004C + DIB 097X on Avicel, washed pretreated poplar, several pretreated unwashed substrates, untreated DDGS and waste-paper. Concentrations of total fermentation products (ethanol+acetate+lactate) formed by co-cultures were in general significantly higher than those reached by single non-cellulolytic strains, indicating cellulose utilization by the co-cultures. On the other hand, the co-cultures produced significantly more ethanol (22 to 63 mol%) compared to the single cellulolytic strain DIB 004C (7 to 13 mol%). Concentrations of ethanol in co-cultures on different substrates were 2.0- to 8.2-times higher than the concentrations of ethanol produced by single cellulolytic strains and generally higher than the concentrations reached by single non-cellulolytic strains (Table 2 and Additional file 1: Table S3). Therefore, the tested co-cultures fermented cellulose and lignocellulosic substrates better than single strains and formed significant amounts of ethanol.

To improve fermentation of cellulose to ethanol, we have investigated 11 different compositions of co-cultures employing isolated *Caldicellulosiruptor* and *Thermoanaerobacter* strains. Co-cultures of *C. saccharolyticus* DSM 8903 and *T. mathranii* DSM 11426 were used as a control. The cultures were grown on 10 g/l Avicel in flasks with shaking without pH control. All tested co-cultures displayed good growth on Avicel but differed in ethanol production. The best ethanol productivity was demonstrated by a co-culture of *Caldicellulosiruptor* DIB 087C and *Thermoanaerobacter* DIB 097X with ethanol comprising 34.8 mol% of the total organic products (Figure 3A and B). This co-culture displayed significant increase of ethanol production compared to the monoculture of *Caldicellulosiruptor* DIB 087C (11.2 mol% ethanol) (Figure 3A and B) and showed higher ethanol production than the co-cultures DIB 004C + DIB 004G (22.1 mol%, Table 2), DIB 004C + DIB 097X (25.4 mol%, Table 2) and other co-cultures tested. Only low ethanol production from Avicel (14.7 mol% ethanol) was achieved with a co-culture of *C. saccharolyticus* DSM 8903 and ethanologenic *T. mathranii* DSM 11426 (Figure 3C). The dependence of ethanol production levels on the composition of co-cultures suggests different interactions of the investigated cellulolytic and non-cellulolytic bacteria in each of the dual co-cultures.

### Growth of single cultures and co-cultures in fermentors with pH control

All growth experiments described above were performed in Hungate tubes or flasks without pH control. Cultivations in fermentors with pH control were conducted with *Caldicellulosiruptor* DIB 004C, *Thermoanaerobacter* DIB 004G and co-cultures of DIB 004C + DIB 004G.

Surprisingly, *Caldicellulosiruptor* DIB 004C produced high amounts of ethanol in fermentors at a constant pH of 6.75: 29.9 mM (35.3 mol%) to 51.3 mM (29.7 mol%) on 20 g/l Avicel; 53.0 mM (31.6 mol%) to 71.5 mM (41.7 mol%) on 100 g/l Avicel; 76.2 mM (64.2 mol%) on 50 g/l glucose; 35.4 mM (52.1 mol%) on 100 g/l xylan; 24.3 mM (66.8 mol%) on 20 g/l of unwashed pretreated poplar. In addition, strain DIB 004C produced 12.5 mM ethanol from Avicel upon cultivation in flasks on media containing 10 g/l MOPS buffer for pH stabilization. Such ethanol productivity for a *Caldicellulosiruptor* strain is extraordinarily high, because so far formation of only traces of ethanol (up to 2 mM) was reported for known bacteria of the genus *Caldicellulosiruptor* grown without pH control [15,17] or with pH control [16].

As expected, *Thermoanaerobacter* DIB 004G also produced high amounts of ethanol when pH was kept at 6.50 to 6.75: 131.8 mM (51.9 mol%) on 25 g/l glucose; 163.5 mM (59.8 mol%) on 50 g/l glucose; 50.7 mM (75.4 mol%) on 20 g/l of unwashed pretreated poplar; 64.3 mM (65.1 mol%) to 120.1 mM (55.0 mol%) on 50 g/l of unwashed pretreated poplar.

**Figure 4:**
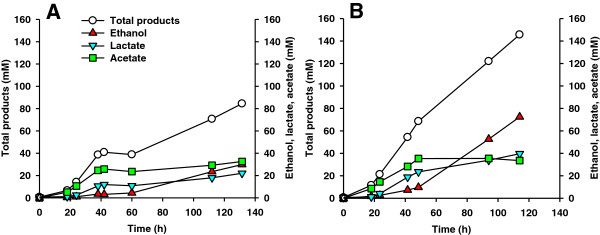
**Fermentation products on Avicel in fermentors. **Cellulolytic strain *Caldicellulosiruptor *sp. DIB 004C (**A**) and a co-culture of DIB 004C and of the non-cellulolytic strain *Thermoanaerobacter *sp. DIB 004G (**B**) were grown on 20 g/l Avicel in fermentors at 72°C with pH stabilized at 6.75.

**Figure 5:**
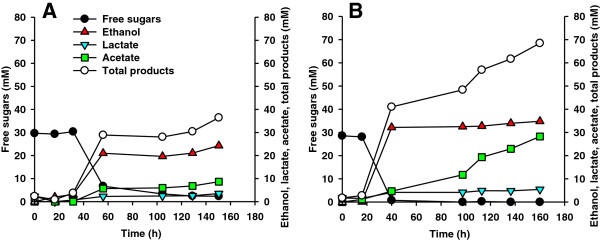
**Fermentation products on pretreated poplar in fermentors. **Cellulolytic strain *Caldicellulosiruptor *sp. DIB 004C (**A**) and a co-culture of DIB 004C and of the non-cellulolytic strain *Thermoanaerobacter *sp. DIB 004G (**B**) were grown on 20 g/l (dry mass) of steam explosion pretreated poplar wood in fermentors at 72°C with pH stabilized at 6.75.

Cultivations in fermentors on Avicel (Figure 4) and pretreated unwashed poplar (Figure 5) with pH control at 6.75 confirmed the functionality of ethanologenic co-cultures. In simultaneously performed fermentations on 20 g/l Avicel, the monoculture of *Caldicellulosiruptor* DIB 004C produced 29.9 mM ethanol and displayed an ethanol:acetate:lactate molar ratio of 1:1.1:0.7 corresponding to an ethanol productivity of 35.7 mol% (Figure 4A). In contrast, the co-culture of *Caldicellulosiruptor* DIB 004C and *Thermoanaerobacter* DIB 004G produced 72.5 mM ethanol as the main fermentation product with an ethanol:acetate:lactate molar ratio of 1:0.5:0.6 and an ethanol yield of 50.0 mol%. Therefore, ethanol production by this co-culture increased 2.4 times compared to the strain DIB 004C alone (Figure 4B).

In simultaneously performed fermentations on 20 g/l of unwashed pretreated poplar, ethanol was the main fermentation product with the monoculture of *Caldicellulosiruptor* DIB 004C (Figure 5A) as well as with the co-culture of *Caldicellulosiruptor* DIB 004C and *Thermoanaerobacter* DIB 004G (Figure 5B). The single culture DIB 004C produced 24.3 mM ethanol (66.8 mol%) and 36.4 mM of total fermentation products (ethanol+acetate+lactate) (Figure 5A). The co-culture of DIB 004C+DIB 004G produced 34.8 mM ethanol (50.9 mol%) and 68.4 mM of total fermentation products. In the first 40 h of growth ethanol was apparently produced by *Thermoanaerobacter* DIB 004G from free sugars present in pretreated poplar. During further growth acetate and small amounts of ethanol and lactate were produced by the co-culture from insoluble carbohydrates (Figure 5B).

Raw poplar used for production of pretreated material contained 42.4% glucan, 11.6% xylan, 1.8% galactan, 2.2% arabinan, 2.6% mannan, 5.4% acid-soluble lignin, 25.0% acid-insoluble lignin, and 0.5% lignin ash, as determined by standard laboratory methods [24]. Although we did not determine the composition of unwashed pretreated poplar, sulfurous acid steam explosion pretreatment of poplar usually results in insignificant loss of approx. 4% of solubilized sugars [25]. Neglecting this loss, the medium with 20 g/l of unwashed pretreated poplar contained at most 52.3 mM glucose, 17.6 mM xylose, 2.2 mM galactose, 3.3 mM arabinose and 3.2 mM mannose equivalents. Assuming a theoretical yield of 2.0 mol of ethanol+acetate+lactate per mol of C6 sugars and 1.67 mol of ethanol+acetate+lactate per mol of C5 sugars, formation of 150.3 mM ethanol+acetate+lactate could be expected from complete fermentation of carbohydrates in the medium containing 20 g/l of employed pretreated poplar. Therefore, 24.2% and 45.5% of fermentable carbohydrates in unwashed pretreated poplar were utilized within the time frame of fermentations by the single strain DIB 004C and the co-culture of DIB 004C+DIB 004G, respectively.

### Fermentation balances

The fermentation balances on glucose (10 g/l), Avicel (10 g/l), washed pretreated poplar (6.05 g/l) and washed pretreated miscanthus (6.09 g/l) for *Caldicellulosiruptor* sp. DIB 004C, *Thermoanaerobacter* sp. DIB 097X and a co-culture of DIB 004C + DIB 097X grown to the stationary phase in flasks without pH control revealed carbon and electron recoveries close to 100% (Table 3). Only ethanol, acetate, lactate, CO_2_ and H_2_ were found as fermentation products. Based on composition of the washed pretreated substrates (Table 3), utilization of fermentable carbohydrates reached 52% and 57% for the co-culture grown on poplar and miscanthus, respectively. It corresponded to a conversion of 30% and 33% of the biomass dry weight, respectively.

**Table 3:** **Fermentation balances of carbohydrates by *Caldicellulosiruptor *sp. str. DIB 004C, *Thermoanaerobacter *sp. strain DIB 097X and co-cultures of the strains DIB 004C and DIB 097X on glucose, Avicel, washed pretreated poplar and washed pretreated miscanthus**

**Growth on glucose**																	
Culture	Glucose initial	Glucose consumed	Ethanol	Acetate	Lactate	CO_2_	H_2_	Cell dry weight	Cell dry weight	Carbon in consumed glucose	Carbon found in products and cells	Carbon recovery	Electron recovery				
	mM	mM	mM	mM	mM	mM	mM	mM carbon	g/l	mM	mM	%	%				
Strain DIB 004C	55.51	30.6 ± 2.8	5.5 ± 1.0	21.9 ± 2.6	35.3 ± 5.5	8.8 ± 7.4	20.8 ± 12.1	9.7 ± 1.9	0.25 ± 0.05	183.8 ± 16.9	179.3 ± 20.2	98.9 ± 18.2	103.8 ± 18.3				
Strain DIB 097X	55.51	55.4 ± 0.4	53.1 ± 7.6	5.3 ± 0.6	55.6 ± 4.9	9.5 ± 13.3	2.4 ± 1.3	14.4 ± 0.1	0.37 ± 0.00	332.0 ± 2.2	307.4 ± 29.5	92.5 ± 8.5	107.9 ± 10.7				
**Growth on Avicel**																	
Culture	Avicel initial	Avicel final	Avicel consumed	Glucose equivalents initial	Glucose equivalents consumed	Ethanol	Acetate	Lactate	CO_2_	H_2_	Cell dry weight	Cell dry weight	Carbon in consumed glucose equivalents	Carbon found in products and cells	Carbon recovery	Electron recovery	Consumption of cellulose in Avicel (100% = 61,7 mM glucose equivalents consumed)
	g/l	g/l	g/l	mM	mM	mM	mM	mM	mM	mM	mM carbon	g/l	mM	mM	%	%	%
Strain DIB 004C	10.00	5.88 ± 0.02	4.12 ± 0.02	61.68	25.4 ± 0.1	4.8 ± 0.9	11.6 ± 2.1	30.4 ± 4.8	9.1 ± 6.3	10 ± 3.4	9.3 ± 1.3	0.24 ± 0.03	152.6 ± 0.6	142.3 ± 17.6	93.2 ± 11.5	103.0 ± 10.3	41.2 ± 0.2
Strain DIB 004C + strain DIB 097X	10.00	6.34 ± 0.02	3.66 ± 0.02	61.68	22.6 ± 0.1	6.2 ± 0.4	10.3 ± 1.9	25.7 ± 3.8	12.6 ± 3.6	9.2 ± 2.4	7.5 ± 1.1	0.19 ± 0.03	135.4 ± 0.6	130.1 ± 14.7	96.1 ± 10.8	105.0 ± 2.6	36.6 ± 0.2
**Growth on washed pretreated poplar**																	
Culture	Poplar initial	Poplar final	Poplar consumed	Glucose equivalents initial	Glucose equivalents consumed	Ethanol	Acetate	Lactate	CO_2_	H_2_	Cell dry weight	Cell dry weight	Carbon in consumed glucose equivalents	Carbon found in products and cells	Carbon recovery	Electron recovery	Consumption of cellulose in poplar (100% = 21.7 mM glucose equivalents consumed)
	g/l	g/l	g/l	mM	mM	mM	mM	mM	mM	mM	mM carbon	g/l	mM	mM	%	%	%
Strain DIB 004C	6.05	4.80 ± 0.43	1.48 ± 0.76	21.70	9.1 ± 4.7	0.2 ± 0.2	13.3 ± 2.2	7.5 ± 0.6	5.1 ± 4.3	8.1 ± 3.1	11.6 ± 0.0	0.30 ± 0.0	54.7 ± 28.0	65.9 ± 6.6	120.6 ± 12.0	137.8 ± 58.1	42.0 ± 21.5
Strain DIB 004C+ strain DIB 097X	6.05	4.21 ± 0.57	1.84 ± 0.57	21.70	11.3 ± 3.5	3.2 ± 1.9	13.2 ± 3.3	6.3 ± 4.0	5.7 ± 5.0	11.0 ± 4.7	10.2 ± 0.1	0.26 ± 0.0	68.0 ± 21.0	67.5 ± 16.8	99.3 ± 24.8	106.5 ± 24.4	52.2 ± 16.2
**Growth on washed pretreated miscanthus**																	
Culture	Miscanthus initial	Miscanthus final	Miscanthus consumed	Glucose equivalents initial	Glucose equivalents consumed	Ethanol	Acetate	Lactate	CO_2_	H_2_	Cell dry weight	Cell dry weight	Carbon in consumed glucose equivalents	Carbon found in products and cells	Carbon recovery	Electron recovery	Consumption of cellulose in miscanthus (100% = 21.8 mM glucose equivalents consumed)
	g/l	g/l	g/l	mM	mM	mM	mM	mM	mM	mM	mM carbon	g/l	mM	mM	%	%	%
Strain DIB 004C	6.09	4.14 ± 0.03	1.95 ± 0.03	21.80	12.0 ± 0.2	2.7 ± 2.3	11.6 ± 1.9	6.3 ± 1.3	4.4 ± 4.7	7.2 ± 5.7	8.9 ± 1.7	0.23 ± 0.04	72.1 ± 1.2	64.6 ± 10.8	89.6 ± 15.0	94.6 ± 17.8	55.1 ± 0.9
Strain DIB 004C + strain DIB 097X	6.09	4.08 ± 0.08	2.01 ± 0.08	21.80	12.4 ± 0.5	5.2 ± 1.8	13.0 ± 1.5	5.5 ± 1.0	4.0 ± 1.9	9.8 ± 1.9	8.4 ± 2.1	0.22 ± 0.05	74.5 ± 2.9	69.2 ± 4.6	92.9 ± 6.1	102.3 ± 6.4	57.0 ± 2.2

Fermentations were performed in 240 ml flasks containing 100 ml cultures with glucose, Avicel, washed pretreated poplar or washed pretreated miscanthus as substrates. Cultures were incubated at 72°C and 100 rpm for 41 h (glucose), 64 h (Avicel) and 400 h (poplar and miscanthus). Initial substrate concentrations were: 10 g/l (55.5 mM) of glucose; 10 g/l (61.7 mM glucose equivalents) of Avicel; 6.05 g/l dry material (21.7 mM glucose equivalents, 00.0 mM xylose equivalents, 0.1 mM galactose equivalents) of washed poplar; 6.09 g/l dry material (21.8 mM glucose equivalents, 1.1 mM xylose equivalents, 0.0 mM galactose equivalents) of washed miscanthus. Concentrations of carbohydrates in washed pretreated poplar and miscanthus were determined according to the standard procedure from NREL [24].

Carbon balances presented in Table 3 were determined by measurements of initial and final carbohydrate concentrations and final carbon-containing end products, including cell dry weight by using the general empirical formula for cell composition of CH_2_N_0.25_O_0.5_[19,32]. For washed pretreated poplar and miscanthus the loss of weight was attributed to the consumption of cellulose, since no xylose was detected in these substrates. Carbon contained in yeast extract and extracellular protein was not included in the carbon recovery. Carbon and electron balances were calculated as described [32].

Data represent averages of the results of six replicate fermentation experiments.

## Discussion

The objective of this study was to isolate extremely thermophilic bacteria suitable for a single-step conversion of lignocellulosic biomass to ethanol at temperatures >70°C. Cellulolytic ethanologenic enrichments growing at 72°C and producing ethanol as the main fermentation product from crystalline cellulose and pretreated poplar wood were obtained from various environmental samples collected in Germany (Additional file 1: Table S1). From these enrichments seven cellulolytic strains of the genus *Caldicellulosiruptor* (Figure 1) and eight non-cellulolytic strains of the genus *Thermoanaerobacter* (Figure 2) were isolated, capable of growing at 72°C.

All *Caldicellulosiruptor* strains were capable of fermenting crystalline cellulose, xylan, as well as glucose and xylose, making them suitable for the hydrolysis and fermentation of lignocellulosic substrates. All *Thermoanaerobacter* strains fermented glucose and xylose and five strains fermented xylan. Fermentation products of all *Caldicellulosiruptor* and *Thermoanaerobacter* strains included ethanol, lactate, acetate, H_2_ and CO_2_.

When *Caldicellulosiruptor* strains were grown in tubes or flasks without pH control on cellulose, cellobiose, glucose, xylan or xylose, 1–5 mM of ethanol was accumulating in the fermentation (Table 1 and Additional file 1: Table S2). Surprisingly, the strain *Caldicellulosiruptor* DIB 004C produced 12.5 mM ethanol from Avicel in flasks with MOPS buffer for pH stabilization. Ethanol concentrations of 71.5 mM (3.3 g/l) and 76.2 mM (3.5 g/l) were obtained when *Caldicellulosiruptor* DIB 004C was grown on Avicel and glucose, respectively, in pH-controlled fermentors, ethanol being the main product in some fermentation runs. In contrast, all know bacteria of the genus *Caldicellulosiruptor* produced only traces or low concentrations (up to 2 mM) of ethanol in fermentations performed with pH control [16] or without pH control [17,18].

The high ethanol concentrations reported were obtained with the wild-type strain *Caldicellulosiruptor* DIB 004C grown on non-optimized medium under non-optimized cultivation conditions. These values are similar to ethanol levels reported for the most of wild-type strains of the thermophilic cellulolytic bacterium *C. thermocellum*, an extensively researched candidate for thermophilic CBP: strain ATCC 27405 in fermentor, 86.8 mM (4 g/l) ethanol [11]; strain LQRI in flasks, 31.2 mM (1.4 g/l) ethanol [13]; strain DSM 1313 in flasks, 28.2 mM (1.3 g/l) ethanol [3,8]. An exception is the ethanol hyper-producing *C. thermocellum* wild-type strain I-1-B. The strain produced from cellulose 86.8 mM (4 g/l) of ethanol in flasks on optimized medium with 14 g/l yeast extract after 168 hours of fermentation [26] and 512 mM (23.6 g/l) of ethanol in fermentors on optimized medium under optimized fermentation conditions after 156 h [27].

Ethanol was the main fermentation product of five isolated *Thermoanaerobacter* strains grown without pH control on cellobiose, glucose, xylan or xylose. The ethanol concentrations obtained were higher than those with the well known ethanologenic bacterium *T. mathranii*[20] which was used as a control (Table 1 and Additional file 1: Table S2). Up to 164 mM ethanol was accumulated when *Thermoanaerobacter* sp. DIB 004G was grown on glucose with pH control. In this respect the isolated *Thermoanaerobacter* sp. strains were comparable to ethanologenic bacteria of the genus *Thermoanaerobacter* producing ethanol as the main fermentation product, e.g. *T. mathranii* subsp. *mathranii* str. A3 (DSM 11426) capable of producing 20 mM ethanol from xylose [20], *T. ethanolicus* JW 200 (ATCC 31550) producing 78 mM from glucose [28] and *T. thermohydrosulfuricus* strain E100-69 (DSM 567) producing 29 mM from glucose [29].

Cellulolytic strains DIB 004C and DIB 101C grew well on insoluble carbohydrates (mainly cellulose) contained in washed pretreated lignocellulosic substrates. At low substrate concentrations, strain DIB 004C utilized up to 89.1% of insoluble cellulose and hemicellulose present in washed pretreated poplar (Table 2) and up to 97% of insoluble cellulose and hemicellulose present in washed pretreated spruce, corn cobs and corn stalks (Additional file 1: Table S4). The ability of novel *Caldicellulosiruptor* strains to utilize all major carbohydrates from lignocellulosic materials can be attributed to the presence of a large set of extracellular glycoside hydrolases, similar to those found in *C. saccharolyticus*[30,31] and *C. bescii*[32]. At high concentrations of Avicel or pretreated lignocellulosic materials the carbohydrate consumption by cellulolytic strains was not complete. In fermentations without pH control this can be attributed to acidification of the media to pH below 5.0. In pH–controlled fermentations osmotic pressure was shown to affect growth of *T. thermosaccharolyticum*[33]*and C. saccharolyticus*[34].

Similar to *C. bescii*[32], the novel *Caldicellulosiruptor* strains displayed planktonic growth on Avicel and lignocellulosic substrates. Microscopic examinations revealed that most of the cells were not attached to the substrate particles. This is in agreement with formation of extracellular glycoside hydrolases and enabled to follow growth via cell density measurements.

High ethanol production from cellulose and pretreated poplar demonstrated for a number of our extremely thermophilic enrichment cultures could be explained by the synergistic functioning of natural co-cultures of cellulolytic (*Caldicellulosiruptor*) and non-cellulolytic (*Thermoanaerobacter*) bacteria isolated from these enrichments. Fermentations using co-cultures of thermophilic cellulolytic and non-cellulolytic bacteria represent a promising approach for CBP technology and have been investigated at 50°C-60°C with co-cultures of thermophilic *Clostridium thermocellum* and different species of *Thermoanaerobacter* or *Thermoanaerobacterium*[7,8,10-13]. In these co-cultures, faster cellulose degradation and increased ethanol production was observed, explained by removal of free sugars, produced from cellulose by *C. thermocellum*, by the non-cellulolytic bacteria [13].

De-novo constructed dual *Caldicellulosiruptor*-*Thermoanaerobacter* co-cultures revealed up to 8-fold increased ethanol yields compared to the monocultures of *Caldicellulosiruptor* strains (Table 2 and Additional file 1: Table S3). Ethanol production by co-cultures was strongly dependent on their composition. From 11 different compositions of dual co-cultures grown on Avicel in flasks, the highest ethanol production was obtained with a co-culture comprising *Caldicellulosiruptor* DIB 087C and *Thermoanaerobacter* DIB 097X: 13.8 mM ethanol and 34.8 mol% of ethanol fraction within the total organic products (Figure 3). In a control experiment, the co-culture of *C. saccharolyticus* DSM 8903 and *Thermoanaerobacter mathranii* DSM 11426 displayed the lowest ethanol yield from all co-cultures tested: 3.3 mM of ethanol and 14.7 mol% of ethanol fraction within the total organic products (Figure 3). The functionality of co-cultures was also confirmed in pH-controlled fermentations. The ethanol concentration on Avicel in the co-culture of DIB 004C+DIB 004G increased more than 2-fold compared to the monoculture of DIB 004C (Figure 4). Growth of the same cultures on unwashed pretreated poplar revealed a 1.4-fold increase in ethanol levels for the co-culture (Figure 5). Therefore, the established co-cultures operated similar to the original ethanologenic enrichments, a synergistic effect of the bacteria in co-cultures being apparent.

Although the amounts of ethanol produced by novel cellulolytic strains and co-cultures are relatively high compared to other *Caldicellulosiruptor* and *Thermoanaerobacter* species, we are currently working on the optimization of strain performance to maximize ethanol levels. Product profiles as well as conversion of pretreated lignocellulosic materials are addressed by classical strain improvement approaches, targeted genetic engineering of the organisms and improved pretreatment methods. The feasibility of genetic modifications of the novel *Caldicellulosiruptor* and *Thermoanaerobacter* strains is supported by the recent progress in the development of genetic tools for *T. saccharolyticum*[19] and *C. thermocellum*[8] in the projects to produce high ethanol yields and the success in genetic manipulation of *C. bescii*[35,36].

## Conclusions

Here we have shown for the first time that the developed extremely thermophilic co-cultures of *Caldicellulosiruptor* and *Thermoanaerobacter* are capable of efficiently converting C6- and C5-sugars from cellulose and various pretreated lignocellulosic materials into ethanol, lactate and acetate, ethanol being the major fermentation product. No external enzyme additions were required since the appropriate cellulolytic and hemicellulolytic enzymes were provided by cellulolytic/xylanolytic *Caldicellulosiruptor* sp. bacteria and by non-cellulolytic/xylanolytic *Thermoanaerobacter* sp. bacteria. Therefore, these co-cultures are promising for direct fermentation of lignocellulosic biomass to ethanol in a CBP process with operating temperatures above 70°C.

In particular the newly identified *Caldicellulosiruptor* strain DIB 004C provides an unmatched combination of efficient hydrolysis of C5- and C6-sugar polymers derived from lignocellulose, high ethanol production levels and conversion of both C5 and C6 sugars. Therefore, the strain represents an ideal basis for the development of a high temperature lignocellulose to ethanol CBP, either with DIB 004C alone or in a co-culture with one of the newly identified *Thermoanaerobacter* strains.

## Methods

### Bacterial strains

The new isolates of extremely thermophilic anaerobic cellulolytic and saccharolytic non-cellulolytic bacteria were obtained from different soil, mud and compost samples collected in the Rhineland and Cologne area in North Rhine-Westphalia, Germany. *Caldicelulosiruptor saccharolyticus* DSM 8903 and *Thermoanaerobacter mathranii* subsp. *mathranii* DSM 11426 were purchased from the DSMZ collection (Deutsche Sammlung von Mikroorganismen und Zellkulturen GmbH).

### Enrichment, isolation and cultivation

A prereduced medium was used for enrichment cultures and cultivation of isolated strains. The medium contained (per liter deionized water): K_2_HPO_4_, 1.5 g; KH_2_PO_4_, 3 g; MgSO_4_ × 7 H_2_O, 0.3 g; CaCO_3_ × 2 H_2_O, 0.05 g; NH_4_Cl, 1.0 g; NaCl, 0.5 g; NaHCO_3_, 0.5 g; NiCl_2_ × 6 H_2_O, 2 mg; FeSO_4_ × 7 H_2_O, 1 mg; NH_4_Fe(III) citrate, 10 mg; MnSO_4_ × H_2_O, 5 mg; CoCl_2_ × 6 H_2_O, 1 mg; ZnSO_4_ × 7 H_2_O, 1 mg; CuSO_4_ × 5 H_2_O, 0.1 mg; H_3_BO_4_, 0.1 mg; Na_2_MoO_4_ × 2 H_2_O, 0.1 mg; Na_2_SeO_3_ × 5 H_2_O, 0.2 mg; Na_2_WoO_4_ × 2 H_2_O, 0.1 mg; nicotinic acid, 2 mg; cyanocobalamin, 0.25 mg; p-aminobenzoic acid, 0.25 mg; calcium pantothenate, 0.25 mg; thiamine-hydrochloride, 0.25 mg; riboflavin, 0.25 mg; lipoic acid, 0.25 mg; folic acid, 0.1 mg; biotin, 0.1 mg; pyridoxine-hydrochloride, 0.1 mg; yeast extract (Difco), 0.5 g; resazurin, 0.5 mg; Na_2_S × 9 H_2_O, 0.75 g.

The medium was prepared under anaerobic conditions under O_2_-free N_2_ and pH was adjusted to 7.2. The medium was dispensed into flasks and Hungate tubes under N_2_ and autoclaved.

Enrichment medium was inoculated with collected samples and incubated at 72°C in 50 ml flasks with 30 ml medium containing 4.3 g/l of cellulose (strips of filter paper Whatman No.1) and 20 g/l of untreated ground beech wood as substrate.

Cellulolytic strains were isolated by serial dilutions of single-cell colonies in Hungate roll tubes [37] with 30 g/l agar and 5 g/l acid-swollen amorphous cellulose. Non-cellulolytic strains were isolated by repeated serial dilutions in liquid medium with 5 g/l glucose.

Isolated strains were cultivated at 72°C in Hungate tubes or flasks with filter paper, microcrystalline cellulose (Avicel PH-101, Fluka), cellobiose, glucose, xylan from beech wood, xylose, washed or unwashed dilute sulfurous acid steam-explosion-pretreated lignocellulosic materials (poplar, spruce, miscanthus, wheat straw, whole corn plants, corn cobs, corn stalks, sugarcane bagasse, sweet sorghum, cotton stalks), dried distillers grains with solubles (DDGS), and waste paper as substrates. Hungate tubes were incubated without shaking. Flasks were incubated with shaking at 100 rpm.

Growth of bacteria was monitored by analysis of fermentation products and determination of optical density of the cultures (OD_600_). For the separation of cells from insoluble substrates, samples were centrifuged in 2 ml tubes for 20 s at 1.700 g.

In experiments on fermentation balance NaHCO_3_ was omitted from the media and insoluble substrates were separated from cells by centrifugation of the cultures in 50 ml tubes for 60 s at 1.700 g. Cells were washed with 0.9% NaCl and substrates were washed with distilled water. After centrifugation cells and substrates were dried at 90°C for 24 h.

Lignocellulosic substrates were pretreated by acidic steam explosion applying 2% (w/v) sulfurous acid at a temperature of 205°C for 5 min prior to sudden release of pressure. To obtain the insoluble carbohydrate fraction, pretreated substrates were washed three times with water at 72°C. Substrates were suspended in distilled water (100 g dry mass/5 liter) and incubated for 16 h at 72°C with stirring. After filtration under vacuum, substrates were washed two times (each time for 2 h with stirring) with equal volumes of water at 72°C. Washed substrates were removed by filtration, dried at 45°C for 66 h and used for growth experiments. Dry weight of washed and unwashed substrates was determined after drying at 105°C for 24 h.

Composition of raw untreated substrates and washed pretreated substrates was determined according to the laboratory analytical procedure "Determination of structural carbohydrates and lignin in biomass" from National Renewable Energy Laboratory (NREL) [24].

Fermentations were carried out in 2-liter stirred vessel fermentors (Biostat B-DCU, B.Braun / Sartorius AG) with a working volume of 1.2 liter. All vessels were equipped with double jackets for temperature control, two Rushton-type stirrer blades and pH-control loops. In order to maintain a constant pressure throughout the cultivation, vessels were additionally equipped with high-precision blow-off valves, controlling the pressure in the range of 1.3-1.5 bar. The medium as described above was supplemented with Avicel, pretreated poplar wood, xylan or glucose. The medium was set to pH 6.75 by automatic addition of NaOH solution and this value maintained throughout the fermentation run. In order to remove oxygen from the medium, the fermentor vessel was flushed with nitrogen for 1 h at a rate of 1 l/min; then Na_2_S × 9 H_2_O was added as described above while gas flushing was stopped. Each fermentor was inoculated with 100 ml of seed culture prepared as described above for cultivation of a single strain and with 50 ml of each seed culture for co-cultivation of two strains. Cellulolytic seed cultures were grown on 10 g/l Avicel. Non-cellulolytic seed cultures were grown on 5 g/l glucose. A temperature of 72°C was maintained during the entire fermentation run.

### Genomic DNA isolation and 16S rRNA gene sequence analysis

DNA from the isolated cultures was extracted using a peqGold Bacterial DNA kit (PEQLAB Biotechnologie GmbH) or a MasterPure DNA Purification Kit (Biozym Scientific GmbH). The DNA was then amplified by PCR employing a bacterial domain-specific primer set for 16S rRNA, 27 forward and 1492 reverse [38], and KOD Hot Start DNA polymerase (Novagen). PCR was carried out according to protocol recommended for KOD Hot Start DNA polymerase by the manufacturer. PCR products were purified with a QIAquick PCR Purification kit (Qiagen) and sequenced.

The 16S rRNA gene sequences were analyzed using nucleotide to nucleotide BLAST (BLASTN) at NCBI (http://www.ncbi.nlm.nih.gov/blast/). Phylogenetic 16S rRNA gene analyses were performed by the neighbor-joining method [39] using the program Mega 4 [40].

### Analysis of fermentation products

Organic fermentation products ethanol, lactate and acetate were analyzed on a Hitachi Lachrom Elite high-performance liquid chromatography (HPLC) system (Hitachi High Technologies). Metabolites were separated on a Rezex ROA 150×4.6 mm column (Phenomenex) under isocratic temperature (65°C) and flow (0.8 ml/min) conditions in 2.5 mM H_2_SO_4_ and then passed through a refractive index (RI) detector (Hitachi L-2490). Identification was performed by comparison of retention times with standards. H_2_ and CO_2_ were analyzed by gas chromatography with thermal conductivity detection. For analysis of CO_2_ the cultures in flasks were acidified with HCl to lower the pH below 1.4.

Carbon balances were determined by measurement of initial and final carbohydrate concentrations and final carbon-containing end products, including cell dry weight, using the general empirical formula for cell composition of CH_2_N_0.25_O_0.5_[19,41]. For washed pretreated poplar and miscanthus the loss of weight was attributed to the consumption of cellulose, since no xylose was detected in these substrates. Carbon contained in yeast extract and extracellular protein was not included in the carbon recovery. Carbon and electron balances were calculated as described [41].

## Abbreviations

CBP: Consolidated bioprocessing; Avicel: Crystalline cellulose; DDGS: Dried distillers grains with solubles; MOPS: 3-Morpholinopropanesulfonic acid; NREL: National Renewable Energy Laboratory; PCR: Polymerase chain reaction; BLAST: Basic local alignment search tool; HPLC: High performance liquid chromatography; RI: Refractive index.

## Competing interests

All authors are current employees of Direvo Industrial Biotechnology GmbH. Direvo actively develops processes for lignocellulose conversion to fuels and chemicals.

## Authors’ contributions

VS, OK and SC conceived the study and wrote the paper. VS and DF carried out the enrichment work and performed the isolation of cultures and the phylogenetic studies. VS, DF, SK and AS performed the fermentation experiments in flasks/tubes. MP and JS performed experiments in fermentors. AS and NL carried out the analysis of fermentation products. All authors read and approved the final manuscript.

## Supplementary Material

Additional file 1: Table S1 Fermentation products of ethanologenic enrichment cultures. **Table S2. **Fermentation products of cellulolytic and non-cellulolytic strains isolated from ethanologenic enrichment cultures. **Table S3. **Fermentation products of *Caldicellulosiruptor *DIB004C alone and in co-cultures with *Thermoanaerobacter *DIB004G and DIB097X. **Table S4.** Fermentation products of *Caldicellulosiruptor *DIB004C and DIB101C on washed pretreated substrates. Click here for file
