# Supplementary material for: Single-step ethanol production from lignocellulose using novel extremely thermophilic bacteria
Source: Biotechnol Biofuels. 2013 Feb 28;6:31. doi: 10.1186/1754-6834-6-31 (PMC3598825; doi:10.1186/1754-6834-6-31)
Supplement: Additional file 1: Table S1 — Fermentation products of ethanologenic enrichment cultures. Table S2. Fermentation products of cellulolytic and non-cellulolytic strains isolated from ethanologenic enrichment cultures. Table S3. Fermentation products of Caldicellulosiruptor DIB004C alone and in co-cultures with Thermoanaerobacter DIB004G and DIB097X. Table S4. Fermentation products of Caldicellulosiruptor DIB004C and DIB101C on washed pretreated substrates. [file 1754-6834-6-31-S1.docx]

## Manuscript Svetlitchnyi et al., Manuscript ID: 1556026388766251

## Table S1 - Fermentation products of ethanologenic enrichment cultures

| Enrichment cultures | Growth on filter paper (4.3 g/l) | | | |  | Growth on pretreated poplar (20 g/l dry weight) | | | |
| --- | --- | --- | --- | --- | --- | --- | --- | --- | --- |
|  | Ethanol  mM | Acetate  mM | Lactate  mM | Ethanol:Acetate:Lactate  mM:mM:mM |  | Ethanol  mM | Acetate  mM | Lactate  mM | Ethanol:Acetate:Lactate  mM:mM:mM |
| #4 | 11.8 ± 0.6 | 12.9 ± 1.0 | 16.3 ± 0.5 | 1 : 1.1 : 1.4 |  | 20.7 ± 0.0 | 6.3 ± 0.1 | 3.4 ± 0.0 | 1 : 0.3 : 0.2 |
| #26 | 10.7 ± 1.1 | 9.3 ± 0.7 | 17.9 ± 0.8 | 1 : 0.9 : 1.8 |  | 8.8 ± 0.3 | 13.0 ± 0.4 | 9.4 ± 0.1 | 1 : 1.5 : 1.1 |
| #28 | 9.1 ± 0.7 | 7.6 ± 0.4 | 17.6 ± 0.2 | 1 : 0.8 : 1.8 |  | 5.4 ± 0.4 | 6.8 ± 0.2 | 4.4 ± 0.8 | 1 : 1.3 : 0.8 |
| #41 | 3.0 ± 0.1 | 11.4 ± 0.0 | 14.6 ± 0.1 | 1 : 3.8 : 4.9 |  | 1.8 ± 0.5 | 11.5 ± 0.2 | 10.3 ± 0.0 | 1 : 6.4 : 5.7 |
| #77 | 8.1 ± 0.1 | 13.0 ± 0.8 | 14.3 ± 0.4 | 1 : 1.6 : 1.8 |  | 8.6 ± 0.9 | 6.9 ± 0.1 | 5.3 ± 0.1 | 1 : 0.8 : 0.6 |
| #87 | 4.9 ± 0.2 | 16.2 ± 0.2 | 10.5 ± 0.2 | 1 : 3.3 : 2.1 |  | 5.9 ± 0.2 | 10.4 ± 0.4 | 10.0 ± 0.3 | 1 : 1.8 : 1.7 |
| #97 | 0.6 ± 0.8 | 1.8 ± 0.1 | 0.1 ± 0.1 | 1 : 3.2 : 0.1 |  | 17.4 ± 0.1 | 8.3 ± 0.1 | 1.9 ± 0.0 | 1 : 0.5 : 0.1 |
| #101 | 13.7 ± 0.9 | 14.4 ± 1.1 | 12.3 ± 0.9 | 1 : 1.0 : 0.9 |  | 11.9 ± 0.5 | 12.2 ± 0.5 | 7.7 ± 0.0 | 1 : 1.0 : 0.6 |
| #103 | 8.7 ± 1.0 | 7.6 ± 0.1 | 16.3 ± 0.5 | 1 : 0.9 : 2.0 |  | 12.6 ± 0.3 | 3.2 ± 1.4 | 2.0 ± 0.6 | 1 : 0.3 : 0.2 |
| #104 | 7.1 ± 0.1 | 10.5 ± 0.0 | 15.9 ± 0.2 | 1 : 1.5 : 2.3 |  | 9.9 ± 1.0 | 9.9 ± 1.9 | 8.6 ± 0.0 | 1 : 1.0 : 0.9 |
| #107 | 4.9 ± 0.9 | 12.7 ± 0.0 | 15.4 ± 0.8 | 1 : 2.6 : 3.6 |  | 17.1 ± 0.1 | 9.2 ± 1.0 | 7.6 ± 0.1 | 1 : 0.5 : 0.4 |

## Cultures were grown at 72°C for 6 days in Hungate tubes on filter paper without shaking and in flasks on unwashed pretreated poplar with shaking at 100 rpm. Experiments were performed in duplicates.

## Table S2 - Fermentation products of cellulolytic and non-cellulolytic strains isolated from ethanologenic enrichment cultures

| Enrichment cultures used for isolation | Substrate | Isolated cellulolytic *Caldicellulosiruptor* sp. strains | | | | |  | Isolated non-cellulolytic *Thermoanaerobacter* sp. strains | | | | |
| --- | --- | --- | --- | --- | --- | --- | --- | --- | --- | --- | --- | --- |
|  |  | Strain | Ethanol | Acetate  mM | Lactate  mM | Ethanol:Acetate:Lactate  mM:mM:mM |  | Strain | Ethanol  mM | Acetate  mM | Lactate  mM | Ethanol:Acetate:Lactate  mM:mM:mM |
|  |  |  | mM |  |  |  |  |  |  |  |  |  |
| #41 | Cellulose (26 mM gluc.equiv.) | DIB041C | 2.7 ± 0.0 | 10.2 ± 0.6 | 14.3 ± 0.0 | 1 : 3.7 : 5.2 |  | no non-cellulolytic strains isolated | | | | |
|  | Cellobiose (25 mM gluc. equiv.) |  | 1.7 ± 0.1 | 8.4 ± 0.8 | 15.1 ± 1.5 | 1 : 5.1 : 9.1 |  |  |  |  |  |  |
|  | Glucose (25 mM) |  | 1.2 ± 0.2 | 8.5 ± 0.4 | 17.6 ± 0.5 | 1 : 6.9 : 14.1 |  |  |  |  |  |  |
|  | Xylan (31 mM xylose equiv.) |  | 1.4 ± 0.5 | 9.5 ± 0.5 | 17.4 ± 1.8 | 1 : 6.7 : 12.3 |  |  |  |  |  |  |
|  | Xylose (30 mM) |  | 1.3 ± 0.1 | 9.1 ± 0.5 | 17.5 ± 0.1 | 1 : 7.3 : 13.9 |  |  |  |  |  |  |
| #87 | Cellulose (26 mM gluc.equiv.) | DIB087C | 1.9 ± 0.1 | 12.9 ± 0.1 | 7.4 ± 0.0 | 1 : 6.8 : 3.9 |  | DIB087G | no growth on cellulose | | | |
|  | Cellobiose (25 mM gluc. equiv.) |  | 1.6 ± 0.0 | 10.8 ± 0.6 | 8.1 ± 0.1 | 1 : 6.8 : 5.1 |  |  | 6.8 ± 1.6 | 9.5 ± 0.4 | 25.8 ± 0.4 | 1 : 1.4 : 3.8 |
|  | Glucose (25 mM) |  | 1.8 ± 0.2 | 14.4 ± 0.3 | 7.1 ± 0.1 | 1 : 8.1 : 4.0 |  |  | 1.2 ± 0.0 | 4.2 ± 0.3 | 23.8 ± 0.0 | 1 : 3.6 : 20.6 |
|  | Xylan (31 mM xylose equiv.) |  | 1.4 ± 0.3 | 13.7 ± 2.1 | 8.3 ± 2.7 | 1 : 10.0 : 6.0 |  |  | no growth on xylan | | | |
|  | Xylose (30 mM) |  | 1.9 ± 0.1 | 14.5 ± 0.1 | 7.8 ± 0.3 | 1 : 7.8 : 4.2 |  |  | 2.9 ± 0.2 | 5.8 ± 0.5 | 17.5 ± 0.7 | 1 : 2.0 : 6.0 |
| #101 | Cellulose (26 mM gluc.equiv.) | for further details see the main Table 1 | | | | |  | DIB101G | no growth on cellulose | | | |
|  | Cellobiose (25 mM gluc. equiv.) |  |  |  |  |  |  |  | 15.4 ± 2.8 | 1.8 ± 0.2 | 9.3 ± 0.5 | 1 : 0.1 : 0.6 |
|  | Glucose (25 mM) |  |  |  |  |  |  |  | 16.3 ± 0.6 | 0.5 ± 0.0 | 12.2 ± 1.8 | 1 : 0.0 : 0.8 |
|  | Xylan (31 mM xylose equiv.) |  |  |  |  |  |  |  | no growth on xylan | | | |
|  | Xylose (30 mM) |  |  |  |  |  |  |  | 15.4 ± 0.0 | 0.8 ± 0.5 | 9.1 ± 1.6 | 1 : 0.1 : 0.6 |
|  | Cellulose (26 mM gluc.equiv.) |  | | | | |  | DIB101X | no growth on cellulose | | | |
|  | Cellobiose (25 mM gluc. equiv.) |  |  |  |  |  |  |  | 12.3 ± 1.4 | 2.7 ± 0.7 | 6.7 ± 0.1 | 1 : 0.2 : 0.5 |
|  | Glucose (25 mM) |  |  |  |  |  |  |  | 9.2 ± 0.9 | 3.7 ± 1.4 | 9.3 ± 1.8 | 1 : 0.4 : 1.0 |
|  | Xylan (31 mM xylose equiv.) |  |  |  |  |  |  |  | 23.3 ± 0.0 | 6.6 ± 0.0 | 3.1 ± 0.3 | 1 : 0.3 : 0.1 |
|  | Xylose (30 mM) |  |  |  |  |  |  |  | 19.8 ± 0.1 | 3.9 ± 0.0 | 6.9 ± 1.0 | 1 : 0.2 : 0.3 |
| #103 | Cellulose (26 mM gluc.equiv.) | DIB103C | 2.0 ± 0.6 | 7.1 ± 0.3 | 15.3 ± 0.0 | 1 : 3.5 : 7.5 |  | DIB103X | no growth on cellulose | | | |
|  | Cellobiose (25 mM gluc. equiv.) |  | 1.1 ± 0.1 | 6.8 ± 1.3 | 15.7 ± 0.4 | 1 : 6.0 : 13.9 |  |  | 36.7 ± 0.1 | 6.6 ± 0.0 | 9.9 ± 0.0 | 1 : 0.2 : 0.3 |
|  | Glucose (25 mM) |  | 1.1 ± 0.0 | 6.7 ± 0.1 | 17.5 ± 0.5 | 1 : 6.0 : 15.6 |  |  | 29.2 ± 0.5 | 0.7 ± 0.3 | 7.8 ± 0.1 | 1 : 0.0 : 0.3 |
|  | Xylan (31 mM xylose equiv.) |  | 1.1 ± 0.1 | 6.2 ± 0.3 | 15.9 ± 0.3 | 1 : 5.4 :14.0 |  |  | 27.4 ± 15.1 | 6.7 ± 0.4 | 2.8 ± 1.2 | 1 : 0.2 : 0.1 |
|  | Xylose (30 mM) |  | 1.0 ± 0.1 | 7.3 ± 0.6 | 16.1 ± 1.4 | 1 : 7.4 : 16.4 |  |  | 25.5 ± 2.1 | 3.8 ± 0.0 | 6.3 ± 0.4 | 1 : 0.2 : 0.2 |
| #104 | Cellulose (26 mM gluc.equiv.) | DIB104C | 2.1 ± 0.2 | 7.8 ± 0.0 | 16.3 ± 0.3 | 1 : 3.7 : 7.6 |  | DIB104X | no growth on cellulose | | | |
|  | Cellobiose (25 mM gluc. equiv.) |  | 1.6 ± 0.1 | 8.6 ± 0.4 | 16.6 ± 0.5 | 1 : 5.3 : 10.3 |  |  | 17.4 ± 0.5 | 9.6 ± 0.3 | 14.1 ± 0.4 | 1 : 0.6 : 0.8 |
|  | Glucose (25 mM) |  | 1.7 ± 0.2 | 8.1 ± 0.5 | 16.7 ± 1.1 | 1 : 4.9 : 10.1 |  |  | 6.7 ± 0.7 | 3.2 ± 0.1 | 23.2 ± 0.0 | 1 : 0.5 : 3.5 |
|  | Xylan (31 mM xylose equiv.) |  | 1.4 ± 0.0 | 9.7 ± 0.1 | 15.1 ± 0.5 | 1 : 7.1 : 11.0 |  |  | weak growth on xylan | | | |
|  | Xylose (30 mM) |  | 1.4 ± 0.3 | 10.1 ± 0.4 | 12.9 ± 0.3 | 1 : 7.3 : 9.2 |  |  | 13.5 ± 0.3 | 5.7 ± 0.8 | 14.2 ± 3.1 | 1 : 0.4 : 1.0 |
| #107 | Cellulose (26 mM gluc.equiv.) | DIB107C | 2.4 ± 0.0 | 9.5 ± 0.4 | 14.2 ± 0.2 | 1 : 4.1 : 6.1 |  | DIB107X | no growth on cellulose | | | |
|  | Cellobiose (25 mM gluc. equiv.) |  | 1.6 ± 0.2 | 6.4 ± 1.1 | 17.4 ± 0.7 | 1 : 3.9 : 10.7 |  |  | 35.9 ± 0.5 | 4.7 ± 0.2 | 12.1 ± 0.5 | 1 : 0.1 : 0.3 |
|  | Glucose (25 mM) |  | 1.8 ± 0.2 | 9.3 ± 0.0 | 14.1 ± 0.2 | 1 : 5.3 : 8.0 |  |  | 22.0 ± 2.9 | 1.9 ± 0.2 | 15.1 ± 1.6 | 1 : 0.1 : 0.7 |
|  | Xylan (31 mM xylose equiv.) |  | 1.3 ± 0.3 | 10.6 ± 0.2 | 13.8 ± 0.0 | 1 : 8.1 : 10.5 |  |  | 24.5 ± 1.5 | 6.1 ± 0.2 | 2.3 ± 0.1 | 1 : 0.3 : 0.1 |
|  | Xylose (30 mM) |  | 1.2 ± 0.2 | 9.7 ± 0.3 | 12.6 ± 0.1 | 1 : 7.8 : 10.1 |  |  | 28.7 ± 3.4 | 4.7 ± 0.7 | 8.5 ± 0.5 | 1 : 0.2 : 0.3 |
| Control strains | Cellulose (26 mM gluc.equiv.) | *Caldicellulosiruptor*  *saccharolyticus*  DSM 8903 | 1.4 ± 0.3 | 5.7 ± 0.1 | 6.6 ± 0.1 | 1 : 4.1 : 4.7 |  | *Thermoanaerobacter*  *mathranii*  DSM11426 | no growth on cellulose | | | |
|  | Cellobiose (25 mM gluc. equiv.) |  | 4.7 ± 0.3 | 7.1 ± 1.5 | 14.4 ± 2.0 | 1 : 1.5 : 3.0 |  |  | 12.4 ± 0.3 | 7.2 ± 0.5 | 18.7 ± 1.2 | 1 : 0.6 : 1.5 |
|  | Glucose (25 mM) |  | 1.7 ± 0.2 | 7.8 ± 1.1 | 14.2 ± 3.7 | 1 : 4.7 : 8.6 |  |  | 17.2 ± 0.4 | 8.5 ± 0.5 | 17.0 ± 1.3 | 1 : 0.5 : 1.0 |
|  | Xylan (31 mM xylose equiv.) |  | 1.8 ± 0.3 | 10.2 ± 0.4 | 7.7 ± 0.3 | 1 : 5.5 : 4.2 |  |  | 19.9 ± 0.7 | 4.9 ± 0.4 | 2.7 ± 0.0 | 1 : 0.2 : 0.1 |
|  | Xylose (30 mM) |  | 2.9 ± 0.5 | 4.1 ± 0.8 | 14.7 ± 1.3 | 1 : 1.4 : 5.1 |  |  | 13.7 ± 0.0 | 4.9 ± 0.1 | 12.6 ± 0.6 | 1 : 0.4 : 0.9 |

## Cultures were grown at 72°C for 6 days in Hungate tubes without shaking. Growth experiments were performed in duplicates.

## Table S3 - Fermentation products of *Caldicellulosiruptor* DIB004C alone and in co-cultures with *Thermoanaerobacter* DIB004G and DIB097X

| Culture | Substrate | Fermentation products | | | | | |
| --- | --- | --- | --- | --- | --- | --- | --- |
|  |  | Ethanol  mM | Acetate  mM | Lactate  mM | Ethanol:Acetate:Lactate  mM:mM:mM | Total products  mM | Ethanol yield  mol% |
| DIB004C | unwashed pretreated  miscanthus (10 g/l) | 3.4 ± 0.0 | 16.7 ± 1.1 | 7.6 ± 0.3 | 1 : 4.9 : 2.2 | 27.7 ± 0.8 | 12.3 ± 0.3 |
| DIB004G |  | 5.0 ± 1.4 | 6.9 ± 0.2 | 3.4 ± 0.1 | 1 : 1.4 : 0.7 | 15.3 ± 1.7 | 32.5 ± 5.7 |
| DIB097X |  | 7.2 ± 0.1 | 7.7 ± 0.5 | 0.0 ± 0.0 | 1 : 1.1 : 0.0 | 14.9 ± 0.6 | 48.2 ± 1.4 |
| DIB004C+DIB004G |  | 9.6 ± 0.5 | 17.6 ± 0.6 | 5.4 ± 0.5 | 1 : 1.8 : 0.6 | 32.6 ± 1.5 | 29.4 ± 0.0 |
| DIB004C+DIB097X |  | 12.9 ± 1.7 | 18.6 ± 0.1 | 6.9 ± 0.3 | 1 : 1.4 : 0.5 | 38.4 ± 2.1 | 33.6 ± 2.4 |
| DIB004C | unwashed pretreated sugarcane  bagasse (10 g/l) | 2.3 ± 1.0 | 14.4 ± 0.6 | 9.7 ± 0.2 | 1 : 6.2 : 4.2 | 26.5 ± 1.4 | 8.7 ± 3.4 |
| DIB004G |  | 5.5 ± 0.4 | 10.4 ± 0.1 | 7.4 ± 0.2 | 1 : 1.9 : 1.3 | 23.2 ± 0.3 | 23.6 ± 1.5 |
| DIB097X |  | 12.8 ± 0.5 | 8.2 ± 0.4 | 3.2 ± 0.0 | 1 : 0.6 : 0.3 | 24.2 ± 0.9 | 52.7 ± 0.1 |
| DIB004C+DIB004G |  | 14.5 ± 0.6 | 15.9 ± 0.5 | 8.8 ± 0.6 | 1 : 1.1 : 0.6 | 39.2 ± 0.7 | 37.0 ± 0.9 |
| DIB004C+DIB097X |  | 13.9 ± 0.5 | 17.9 ± 0.1 | 7.9 ± 0.3 | 1 : 1.3 : 0.6 | 39.7 ± 0.7 | 35.2 ± 0.8 |
| DIB004C | unwashed pretreated wheat  straw (10 g/l) | 3.6 ± 0.7 | 16.3 ± 0.7 | 7.1 ± 0.3 | 1 : 4.6 : 2.0 | 27.0 ± 1.1 | 13.3 ± 2.0 |
| DIB004G |  | 5.2 ± 0.1 | 4.4 ± 0.1 | 3.2 ± 0.0 | 1 : 0.8 : 0.6 | 12.7 ± 0.1 | 40.8 ± 0.7 |
| DIB097X |  | 8.0 ± 0.4 | 5.5 ± 0.1 | 0.0 ± 0.0 | 1 : 0.7 : 0.0 | 13.6 ± 0.3 | 59.1 ± 1.7 |
| DIB004C+DIB004G |  | 7.2 ± 0.2 | 6.3 ± 0.2 | 2.5 ± 0.1 | 1 : 0.9 : 0.4 | 16.0 ± 0.5 | 44.9 ± 0.1 |
| DIB004C+DIB097X |  | 9.8 ± 0.3 | 7.2 ± 0.2 | 2.3 ± 0.0 | 1 : 0.7 : 0.2 | 19.3 ± 0.5 | 50.8 ± 0.4 |
| DIB004C | unwashed pretreated corn  stalks (10 g/l) | 3.7 ± 0.1 | 17.4 ± 1.1 | 8.4 ± 0.4 | 1 : 4.7 : 2.3 | 29.5 ± 0.6 | 12.4 ± 0.7 |
| DIB004G |  | 5.0 ± 0.1 | 6.9 ± 0.0 | 3.4 ± 0.0 | 1 : 1.4 : 0.7 | 15.4 ± 0.2 | 32.7 ± 0.2 |
| DIB097X |  | 9.2 ± 1.0 | 7.3 ± 0.1 | 0.0 ± 0.0 | 1 : 0.8 : 0.0 | 16.5 ± 1.1 | 55.9 ± 2.2 |
| DIB004C+DIB004G |  | 11.5 ± 0.5 | 8.4 ± 0.1 | 6.7 ± 0.8 | 1 : 0.7 : 0.6 | 26.5 ± 0.4 | 43.3 ± 2.4 |
| DIB004C+DIB097X |  | 12.7 ± 0.4 | 17.8 ± 0.2 | 7.5 ± 0.1 | 1 : 1.4 : 0.6 | 38.0 ± 0.7 | 33.5 ± 0.5 |
| DIB004C | untreated  DDGS (10 g/l) | 1.4 ± 0.1 | 10.3 ± 0.2 | 3.3 ± 0.4 | 1 : 7.6 : 2.4 | 15.0 ± 0.6 | 9.1 ± 0.0 |
| DIB004G |  | 9.6 ± 0.1 | 4.3 ± 0.1 | 2.5 ± 0.0 | 1 : 0.4 : 0.3 | 16.4 ± 0.2 | 58.7 ± 0.3 |
| DIB097X |  | 10.9 ± 0.1 | 4.6 ± 0.1 | 1.9 ± 0.0 | 1 : 0.4 : 0.2 | 17.4 ± 0.3 | 63.0 ± 0.3 |
| DIB004C+DIB004G |  | 10.4 ± 0.2 | 4.1 ± 0.1 | 2.1 ± 0.0 | 1 : 0.4 : 0.2 | 16.6 ± 0.3 | 62.6 ± 0.0 |
| DIB004C+DIB097X |  | 11.2 ± 0.1 | 4.8 ± 0.5 | 2.0 ± 0.0 | 1 : 0.4 : 0.2 | 18.1 ± 0.6 | 62.0 ± 1.4 |
| DIB004C | untreated  waste-paper (10 g/l) | 2.9 ± 0.2 | 27.3 ± 1.3 | 10.1 ± 0.6 | 1 : 9.3 : 3.4 | 40.4 ± 0.5 | 7.3 ± 0.7 |
| DIB004G |  | 2.6 ± 0.1 | 2.5 ± 0.0 | 2.9 ± 0.2 | 1 : 1.0 : 1.1 | 8.0 ± 0.3 | 32.8 ± 0.1 |
| DIB097X |  | 2.2 ± 0.0 | 2.8 ± 0.2 | 0.0 ± 0.0 | 1 : 1.3 : 0.0 | 5.1 ± 0.2 | 44.0 ± 1.3 |
| DIB004C+DIB004G |  | 6.0 ± 0.3 | 27.3 ± 0.1 | 10.2 ± 0.1 | 1 : 4.6 : 1.7 | 43.4 ± 0.3 | 13.7 ± 0.5 |
| DIB004C+DIB097X |  | 6.8 ± 0.2 | 25.8 ± 0.8 | 10.4 ± 0.4 | 1 : 3.8 : 1.5 | 43.0 ± 0.5 | 15.9 ± 0.2 |

## Cultures were grown at 72°C for 6 days in flasks with shaking at 100 rpm. Growth experiments were performed in duplicates.

**Table S4 - Fermentation products of *Caldicellulosiruptor* DIB004C and DIB101C on washed pretreated substrates**

| Culture | Substrate  (2.9 g dry weight per liter medium) | Maximum expected concentration of ethanol+acetate+lactate at 100% utilization of C5- and C6-sugars in the substrate ^1)^  mM | Fermentation products | | | | | C5- and C6-sugar utilization ^2)^  % of maximum |
| --- | --- | --- | --- | --- | --- | --- | --- | --- |
|  |  |  | Ethanol  mM | Acetate  mM | Lactate  mM | Ethanol:Acetate:Lactate  mM : mM : mM | Total  mM |  |
| DIB 004C | Poplar | 20.9 | 1.5 ± 0.1 | 10.9 ± 0.2 | 6.2 ± 0.8 | 1 : 7.2 : 4.1 | 18.6 ± 0.8 | 89.1 ± 3.9 |
| DIB 101C |  |  | 1.7 ± 0.3 | 11.8 ± 0.2 | 4.6 ± 0.2 | 1 : 6.9 : 2.7 | 18.1 ± 0.7 | 86.7 ± 3.2 |
| DIB 004C | Spruce | 17.1 | 4.1 ± 0.1 | 9.2 ± 0.2 | 3.3 ± 0.6 | 1 : 2.2 : 0.8 | 16.6 ± 0.7 | 97.1 ± 4.3 |
| DIB 101C |  |  | 2.0 ± 0.0 | 11.0 ± 0.3 | 3.0 ± 0.2 | 1 : 5.5 : 1.5 | 16.0 ± 0.4 | 93.8 ± 2.5 |
| DIB 004C | Miscanthus | 21.7 | 0.6 ± 0.1 | 13.8 ± 0.1 | 2.1 ± 0.5 | 1 : 21.5 : 3.2 | 16.5 ± 0.4 | 76.1 ± 1.9 |
| DIB 101C |  |  | 0.9 ± 0.0 | 14.5 ± 1.3 | 1.7 ± 0.7 | 1 : 16.7 : 1.9 | 17.0 ± 0.6 | 78.3 ± 2.9 |
| DIB 004C | Wheat straw | 23.5 | 1.6 ± 0.5 | 12.0 ± 1.0 | 1.0 ± 0.1 | 1 : 7.6 : 0.6 | 14.6 ± 1.6 | 62.1 ± 6.9 |
| DIB 101C |  |  | 1.3 ± 0.1 | 9.8 ± 0.3 | 1.0 ± 0.6 | 1 : 7.4 : 0.7 | 12.1 ± 0.4 | 51.5 ± 1.9 |
| DIB 004C | Whole corn plants | 22.0 | 1.7 ± 1.0 | 14.4 ± 0.4 | 1.3 ± 0.5 | 1 : 8.3 : 0.8 | 17.5 ± 0.8 | 79.4 ± 3.8 |
| DIB 101C |  |  | 0.8 ± 0.0 | 12.3 ± 0.3 | 0.8 ± 0.4 | 1 : 14.9 : 1.0 | 14.0 ± 0.2 | 63.5 ± 0.7 |
| DIB 004C | Corn cobs | 24.2 | 3.6 ± 0.3 | 15.4 ± 1.0 | 4.6 ± 0.2 | 1 : 4.3 : 1.3 | 23.6 ± 1.1 | 97.5 ± 4.7 |
| DIB 101C |  |  | 2.1 ± 0.2 | 15.5 ± 0.7 | 5.3 ± 0.6 | 1 : 7.3 : 2.5 | 22.9 ± 1.2 | 94.8 ± 5.0 |
| DIB 004C | Corn stalks | 22.4 | 2.5 ± 0.3 | 16.7 ± 0.4 | 1.8 ± 0.5 | 1 : 6.8 : 0.7 | 20.9 ± 1.2 | 93.5 ± 5.4 |
| DIB 101C |  |  | 1.7 ± 0.1 | 17.3 ± 0.0 | 0.8 ± 0.0 | 1 : 9.9 : 0.5 | 19.9 ± 0.1 | 88.9 ± 0.3 |
| DIB 004C | Sugarcane bagasse | 21.2 | 3.4 ± 0.6 | 13.9 ± 1.3 | 2.0 ± 0.3 | 1 : 4.1 : 0.6 | 19.2 ± 1.7 | 90.6 ± 8.0 |
| DIB 101C |  |  | 2.1 ± 0.1 | 12.9 ± 0.1 | 1.5 ± 0.8 | 1 : 6.3 : 0.7 | 16.5 ± 0.6 | 77.6 ± 3.0 |
| DIB 004C | Sweet sorghum plants | 22.4 | 1.9 ± 0.2 | 12.7 ± 0.1 | 1.9 ± 1.3 | 1 : 6.8 : 1.0 | 16.4 ± 1.3 | 73.2 ± 6.0 |
| DIB 101C |  |  | 1.3 ± 0.2 | 12.2 ± 0.0 | 0.9 ± 0.2 | 1 : 9.3 : 0.7 | 14.4 ± 0.3 | 64.3 ± 1.4 |
| DIB 004C | Cotton stalks | 17.6 | 0.6 ± 0.1 | 5.7 ± 0.2 | 0.9 ± 0.6 | 1 : 8.9 : 1.4 | 7.2 ± 0.5 | 40.7 ± 2.9 |
| DIB 101C |  |  | 0.5 ± 0.0 | 5.2 ± 0.1 | 0.7 ± 0.4 | 1 : 10.5 : 1.3 | 6.4 ± 0.3 | 36.1 ± 1.7 |

Cultures were grown on washed pretreated substrates (2.9 g/l) at 72°C for 6 days in flasks with shaking at 100 rpm. Growth experiments were performed in duplicates.

^1)^ Calculated assuming maximum formation of 1.67 mol or 2 mol of products (ethanol + acetate + lactate) from 1 mol of C5- or C6-sugar, respectively. Concentrations of glucose, xylose, galactose, arabinose and mannose in pretreated washed substrates were determined according to the standard procedure from NREL [24].

^2)^ Calculated from the ratio between the total concentration of products (ethanol + acetate + lactate) formed upon growth on the respective substrates and the expected concentration of products at 100% utilization of C5- and C6-sugars in the substrate.
